# Addenbrooke's Cognitive Examination and Individual Domain Cut-Off Scores for Discriminating between Different Cognitive Subtypes of Parkinson's Disease

**DOI:** 10.1155/2015/579417

**Published:** 2015-08-17

**Authors:** Dagmar Berankova, Eva Janousova, Martina Mrackova, Ilona Eliasova, Milena Kostalova, Svetlana Skutilova, Irena Rektorova

**Affiliations:** ^1^Applied Neuroscience Research Group, CEITEC MU, Masaryk University, 625 00 Brno, Czech Republic; ^2^Department of Neurology, University Hospital in Ostrava, 708 52 Ostrava, Czech Republic; ^3^Department of Rehabilitation, Faculty of Medicine, University of Ostrava, 708 52 Ostrava, Czech Republic; ^4^Institute of Biostatistics and Analyses, Faculty of Medicine, Masaryk University, 625 00 Brno, Czech Republic; ^5^Department of Neurology, School of Medicine, Masaryk University and St. Anne's Hospital, 656 91 Brno, Czech Republic; ^6^Second Department of Neurology, School of Medicine, Masaryk University and Brno Teaching Hospital, 625 00 Brno, Czech Republic

## Abstract

*Objective*. The main aim of this study was to verify the sensitivity and specificity of Addenbrooke's Cognitive Examination-Revised (ACE-R) in discriminating between Parkinson's disease (PD) with normal cognition (PD-NC) and PD with mild cognitive impairment (PD-MCI) and between PD-MCI and PD with dementia (PD-D). We also evaluated how ACE-R correlates with neuropsychological cognitive tests in PD. *Methods*. We examined three age-matched groups of PD patients diagnosed according to the Movement Disorder Society Task Force criteria: PD-NC, PD-MCI, and PD-D. ROC analysis was used to establish specific cut-off scores of ACE-R and its domains. Correlation analyses were performed between ACE-R and its subtests with relevant neuropsychological tests. *Results*. Statistically significant differences between groups were demonstrated in global ACE-R scores and subscores, except in the language domain. ACE-R cut-off score of 88.5 points discriminated best between PD-MCI and PD-NC (sensitivity 0.68, specificity 0.91); ACE-R of 82.5 points distinguished best between PD-MCI and PD-D (sensitivity 0.70, specificity 0.73). The verbal fluency domain of ACE-R demonstrated the best discrimination between PD-NC and PD-MCI (cut-off score 11.5; sensitivity 0.70, specificity 0.73) while the orientation/attention subscore was best between PD-MCI and PD-D (cut-off score 15.5; sensitivity 0.90, specificity 0.97). ACE-R scores except for ACE-R language correlated with specific cognitive tests of interest.

## 1. Introduction

Parkinson's disease (PD) is considered to be a motor disorder, but nonmotor symptoms have recently attracted more attention as they have a major impact on patient quality of life [1, 2]. The major risk factors for developing dementia associated with PD are higher age, more severe Parkinsonism, postural instability with gait difficulty, and mild cognitive impairment at the time of evaluation. The prevalence of dementia in PD is approximately 30%; the cumulative prevalence reaches up to 80% after 8–10 years of the disease progression [3–5]. PD is often associated with some type of cognitive decline even in the absence of fully blown dementia, and mild cognitive impairment (MCI) is present in about 25% of PD patients [6–8].

MCI is characterized by subjective and objective deterioration of cognitive functions with retention of normal social life and daily functioning [9]. Impaired attention and executive functioning are the most common forms of early cognitive deficit in PD [6, 10]. Deficits in memory, visual spatial skills, and language may also occur, in combination with attentional and executive deficits or alone. Impaired executive functions and posterior cortical deficits may predict the development of dementia later in the course of the disease [10–12]. Criteria for MCI in PD (PD-MCI) were formulated by the Movement Disorder Society (MDS) Task Force in their guidelines for the diagnosis of MCI [6]. PD-MCI was defined as a cognitive decline reported by the patient, carer, or clinician with a performance of one to two standard deviations (SD) below the mean for an age-matched control population on two or more tests from a neuropsychological battery as well as the lack of a confounding cause for poor test performance (e.g., depression). Neuropsychological investigations are quite time-consuming and distressing to patients. It is necessary to have screening instruments to identify PD-MCI and dementia in PD (PD-D). While the Mini Mental State Examination (MMSE) cannot distinguish PD with normal cognition (PD-NC) [13], several screening instruments have been developed or validated for screening PD-MCI [14, 15]. We focused on Addenbrooke's Cognitive Examination-Revised (ACE-R) version and its utility in discriminating between PD-NC and PD-MCI and between PD-MCI and PD-D. In addition to global cut-off scores we aimed at providing cut-off values for all cognitive domains evaluated by this screening instrument.

The ACE-R is a brief cognitive screening battery assessing five neuropsychological domains: orientation and attention (ACE-R OA), memory (ACE-R M), verbal fluency (ACE-R F), language (ACE-R L), and visuospatial abilities (ACE-R VA). It incorporates the widely used MMSE but provides a more thorough assessment of cognitive function. As a screening tool for dementia, it has high reliability and validity, and its utility in a number of neurological conditions has been demonstrated [14–19].

ACE-R was translated into Czech [20] and slightly adapted for ease of use [21]. Normative data exist for healthy elderly Czech people [22]. While several previous studies have employed ACE-R in identifying PD-MCI and PD-D (for review, see [23]), none of the studies has identified cut-off scores for individual ACE-R domains. We examined three age-matched groups of PD patients: PD-NC, PD-MCI, and PD-D. We used receiver-operating curve (ROC) analysis in order to establish the specific cut-off scores for ACE-R and its cognitive domains for discriminating among these three groups. We also evaluated how ACE-R correlates with relevant neuropsychological cognitive tests in PD.

## 2. Materials and Methods

Altogether, 69 patients with PD were enrolled in the study: 22 PD-NC, 37 PD-MCI, and 10 PD-D according to published criteria [5, 6] (Table 1). PD-MCI was defined as a cognitive decline reported by the patient, carer, or clinician, with a performance 1.5 SD below the mean for age-matched control population on two or more tests from the neuropsychological battery (our battery is described in more detail in the text below) as well as the lack of a confounding cause for poor test performance. This is in accordance with level 1 (comprehensive) MDS criteria for diagnosis of PD-MCI [6].

All of the patients were assessed by a clinician and the presence of a current depressive episode excluded subjects from the study. Beck Depression Inventory was used to evaluate depressive symptoms. Unified Parkinson's Disease Rating Scale, part III: Motor Examination (UPDRS III) was employed to evaluate motor symptoms of PD [24].

All of the assessments were conducted when the patients were in their “on” state on dopaminergic medication. Patients were on levodopa ± dopamine agonist ± COMT (catechol-o-methyltransferase) inhibitor. None of the patients were on antipsychotic treatment at the time of examination. All patients with PD-D received cholinesterase inhibitors. None of the included subjects received deep brain stimulation surgery for PD. The study was approved by the local ethics committee, and all of the patients signed an informed consent form.

*Cognitive Assessment Using a Neuropsychological Battery*. To detect cognitive decline in the attention domain, we used selected subtests from the Wechsler Adult Intelligence Scale-Revised (WAIS-R): Digit Span (WAIS-R DS), Coding (WAIS-R C), Arithmetic (WAIS-R A) [23, 25–27], and the Stroop Colour and Word Test, part A, word naming (SCWT A), and part B, colour naming (SCWT B) [28].

To detect memory impairment, we used selected subtests from the Wechsler Memory Scale-III (WMS-III): stories for testing logical memory—immediate recall (LMI), stories—delayed recall (LMD), list of words—immediate recall (LWI), delayed recall (LWD), and recognition of the list of words (LWR) [29].

To evaluate executive functions, we used WAIS-R Similarities (WAIS-R S) [25] and SCWT, part C (SCWT C, i.e., colour-word interference) [28], subtests.

To detect impairment of visuospatial functions, we used the Clock Test (CT) [30] and WAIS-R Picture Completion (WAIS-R PC) [25].

To evaluate language domain, we used the Mississippi Aphasia Screening Test (MAST) [31] and letter verbal fluency (VF) [32].

To achieve our goal, all of the patients were also examined by ACE-R [22]. The maximum ACE-R score (i.e., the best performance) is 100 points. In the ACE-R AO domain, it is possible to achieve a maximum of 18 points; in the ACE-R M domain, the maximum is 26 points; in the ACE-R F domain, it is 14 points; in the ACE-R L domain, it is 26 points; and in the ACE-R VA domain, it is 16 points.

To compare the clinical characteristics of the PD-NC, PD-MCI, and PD-D subjects and their results in ACE-R and its subtests, we used Kruskal-Wallis and Chi-square tests. ROC analysis with AUC (95% CI) was performed and used to evaluate subject performance in ACE-R and its subtests in order to distinguish between PD-MCI and PD-NC and between PD-D and PD-MCI. Sensitivity, specificity, positive predictive value (PPV), and negative predictive value (NPV) were calculated for all possible cut-off values. The cut-off points with the highest Youden index (i.e., the maximum sum of sensitivity and specificity) were selected as the best cut-off point values for discriminating between PD-NC and PD-MCI and between PD-MCI and PD-D.

We also performed correlation analysis between each ACE-R domain and specific neurocognitive tests of interest using nonparametric Spearman's rho coefficient, which was corrected for age (i.e., partial correlation coefficients were calculated). We correlated the ACE-R AO domain (attention, orientation) with WAIS-R C, WAIS-R A, WAIS-R DS, and SCWT—A, B. The ACE-R M domain (memory) was correlated with LMI, LMD, LWI, LWD, and LWR. The ACE-R L domain (language) was correlated with MAST and letter VF. The ACE-R F (executive functions) was correlated with WAIS-R S, WAIS-R PC, and SCWT C. Finally, the ACE-R VA domain (visuospatial abilities) was correlated with WAIS-R PC and CT.

The level of significance was set at *α* = 0.05. Statistical analyses were performed by IBM SPSS Statistics software (version 21) and MATLAB R2010b software.

## 3. Results and Discussion

### 3.1. Results

A comparison of the clinical characteristics of PD-NC, PD-MCI, and PD-D patients reveals that there were no differences between the groups in sex, age, PD duration, or UPDRS III (Table 1). However, there were differences in education and in daily L-dopa dose. The length of education was significantly shorter in PD-D than in PD-NC (the difference in medians is only one year). The L-dopa dose was significantly lower in PD-D than in PD-NC. There were statistically significant differences among the three patient groups in cognitive tests in MMSE, ACE-R, and all the ACE-R subtests with the exception of ACE-R L. Specifically, scores in MMSE and in ACE-R and its subtests were highest in PD-NC and lowest in PD-D.

**Figure 1:**
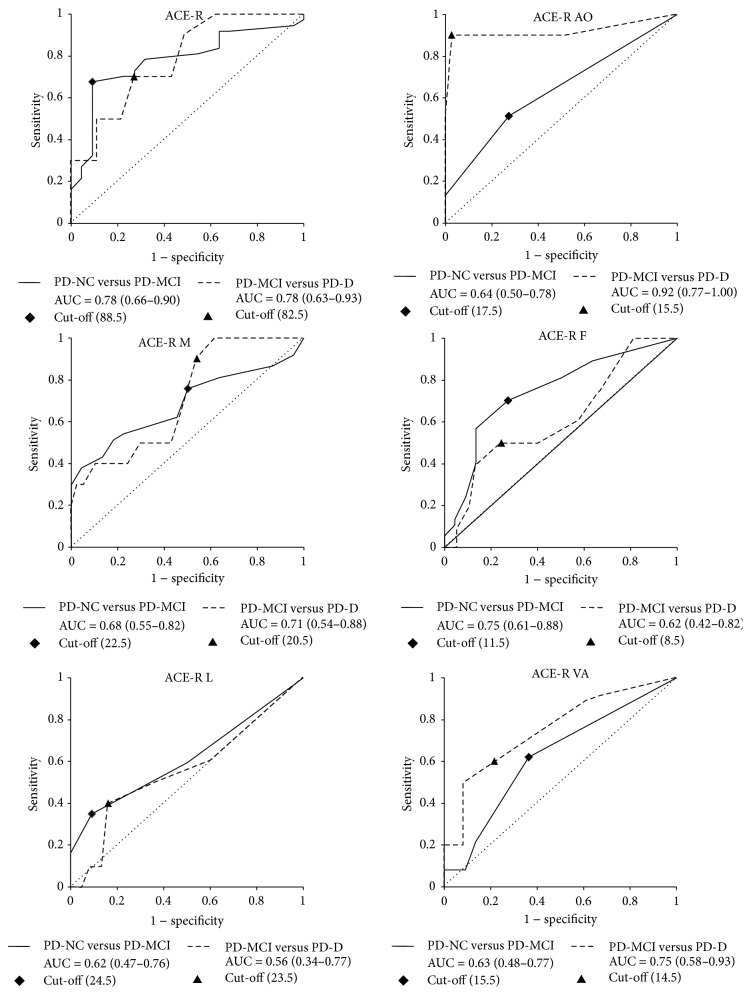
ROC curves distinguishing between patient groups using ACE-R and its subtests. ACE-R OA: orientation and attention domain of ACE-R, ACE-R M: memory domain of ACE-R, ACE-R F: verbal fluency domain of ACE-R, ACE-R L: language domain of ACE-R, ACE-R VA: visuospatial abilities domain of ACE-R, AUC: area under the curve, PD-NC: Parkinson's disease with normal cognition, PD-MCI: Parkinson's disease with mild cognitive impairment, and PD-D: Parkinson's disease with dementia.

Results of ROC analysis including AUC estimates (with 95% confidence intervals) are summarized in Table 2 and visualized via ROC curves in Figure 1. Cut-off scores for global scores of ACE-R and its domains are displayed in Table 2. The ACE-R global cut-off score to differentiate between PD-NC and PD-MCI is 88.5 points (with 0.68 sensitivity and 0.91 specificity) and 82.5 points (with 0.70 sensitivity and 0.73 specificity) to differentiate between PD-MCI and PD-D.

ACE-R and ACE-R M enable discrimination between PD-NC and PD-MCI (with AUC of 0.78 and 0.68, resp.) and between PD-MCI and PD-D (AUC of 0.78 and 0.71, resp.). ACE-R AO and ACE-R VA differentiate between PD-MCI and PD-D (AUC of 0.92 and 0.75, resp.). ACE-R F differentiates between PD-NC and PD-MCI (AUC 0.75). ACE-R L does not enable differentiation among the patient groups (this is shown in Table 1). Table 2 also shows cut-off point estimates based on the Youden index (i.e., the maximum sum of sensitivity and specificity) for ACE-R and its subtests. The cut-off points are also shown in Figure 1.

Partial correlation coefficients between each ACE-R domain and specific neurocognitive tests of interest corrected for patient age are depicted in Table 3. There was no statistically significant correlation between ACE-R AO and WAIS-R C or between ACE-R L and MAST and letter VF. All other correlations were statistically significant.

### 3.2. Discussion

Based on the ROC analysis of ACE-R, the best cut-off score for detecting PD-MCI was 88.5 points with 0.68 sensitivity and 0.91 specificity, with AUC of 0.78 (95% confidence interval (CI) 0.66–0.90). Our result accords well with previous study results in PD-MCI [33] where the authors used the same criteria for PD-MCI diagnosis and demonstrated 0.69 sensitivity and 0.84 specificity with the same ACE-R cut-off score. Komadina et al. (2011) found lower sensitivity (0.61) and specificity (0.64) for higher cut-off scores (93 points) but the authors used different criteria for PD-MCI diagnosis [34]. Our best cut-off score for detecting PD-D was 82.5 points with 0.70 sensitivity and 0.73 specificity, with AUC of 0.78 (95% CI 0.63–0.93). Similar results were found by Biundo and co-workers with a lower cut-off score of 80 points [35], while Reyes et al. (2009) reached higher sensitivity (0.92) and specificity (0.93) with the same cut-off score [15]. These discrepancies could have been caused by the fact that different techniques were used to assess the instrumental and basic activities of daily living. We used a semistructured interview performed with both the patients and their caregivers. A limitation of our study might be the small sample size of the PD-D group.

In addition to cut-offs for the total ACE-R score, our study presents cut-off scores of individual cognitive ACE-R domains for predicting PD-MCI and PD-D which is novel. We also demonstrate for the first time that individual ACE-R domains subscores in PD subjects correlate well with relevant tests scores derived from our comprehensive neuropsychological battery. The verbal fluency domain had the highest sensitivity and specificity for discrimination between PD-NC and PD-MCI with a cut-off score of 11.5 points (sensitivity 0.70, specificity 0.73) and AUC of 0.75 (95% CI 0.61–0.88). The memory domain had a cut-off score of 22.5 points (sensitivity 0.76, specificity 0.50) and AUC of 0.68 (95% CI 0.55–0.82). In a study by Komadina et al. [34] the ACE-R verbal fluency domain was found to be the only domain which was significantly different between PD-NC and PD-MCI [34]. This is in line with our study results. However, Komadina et al. [34] did not use the ROC analysis and the authors used different PD-MCI criteria [34]. Therefore, the two studies cannot be directly compared.

Using MDS criteria for PD-MCI diagnosis, Biundo et al. [35] demonstrated that specific neuropsychological tests evaluating executive functions, memory, and visuospatial functions reached significant screening and diagnostic validity in predicting PD-MCI. Interestingly, Cholerton et al. [36] used detailed neuropsychological examination in PD-MCI and factor analysis to show that the verbal fluency category loaded on two factors: with visuospatial skills and with executive functions. In view of these results, it is not surprising that the verbal fluency domain of ACE-R reached the best diagnostic validity in predicting PD-MCI in our study.

The most sensitive and specific ACE-R domain for discrimination between PD-MCI and PD-D was attention and orientation, with a cut-off score of 15.5 points (sensitivity 0.90, specificity 0.97) and AUC of 0.92 (95% CI 0.77–1.00). This domain significantly correlated with neuropsychological tests of interest evaluating attention and psychomotor speed. In the visuospatial abilities domain, a cut-off score of 14.5 points distinguished between PD-MCI and PD-D with sensitivity of 0.60 and specificity of 0.78. The language domain did not reveal good screening validity in predicting either PD-MCI or PD-D. This could have been caused by the fact that our subjects were normal or only very slightly affected in this domain as well as on the MAST. This result is in line with the published literature showing fewer deficits in the language domain in PD [24, 35].

The ACE-R takes 20–30 minutes and yields quite a lot of information about the global level of cognitive functions and about specific cognitive deficits in PD patients. Our cut-off scores for ACE-R and individual ACE-R domains may help in screening for PD-MCI subjects and in assessing their cognitive profile.

We would particularly like to stress our result regarding the orientation and attention domain alone which had very good screening validity for PD-D prediction. The AUC estimates indicate that this subscore was superior to the total ACE-R score in discriminating PD-MCI from PD-D in our dataset (AUC 0.92 versus 0.78). Therefore, this subtest could be recommended for a quick PD dementia screening. However, the whole ACE-R is needed to assess global cognition and specific cognitive profiles in PD-D subjects.

## 4. Conclusion

While the whole ACE-R is a suitable screening instrument for discriminating among PD-NC, PD-MCI, and PD-D, we also provide for the first time specific ACE-R domains cut-off scores that best distinguish between PD-NC and PD-MCI (verbal fluency and memory domains) and between PD-MCI and PD-D (orientation and attention domain). These parts of ACE-R are easy and quick to administer and may be of help in screening specific PD cognitive subtypes.

## Figures and Tables

**Table 1:** Patient characteristics (*n* = 69). Characteristics are described as median (min-max).

	Total (*n* = 69)	PD-NC (*n* = 22)	PD-MCI (*n* = 37)	PD-D (*n* = 10)	*p* value	Post hoc *p* values		
						PD-NC × PD-MCI	PD-MCI × PD-D	PD-NC × PD-D
Sex, males (%)	41 (59.4%)	13 (59.1%)	25 (67.6%)	3 (30.0%)	0.100			
Age (years)	68 (49–86)	70 (51–86)	67 (49–81)	65 (54–82)	0.754			
Education (years)	13 (9–18)	13 (12–18)	13 (9–18)	12 (9–18)	**0.006**	0.466	0.062	**0.004**
PD duration (years)	7 (1–22)	7 (3–21)	8 (1–18)	6 (2–22)	0.510			
L-dopa dose (mg/day)	907 (0–2275)	1037 (0–2275)	931 (56–2108)	591 (160–1836)	**0.042**	0.870	0.158	**0.044**
UPDRS III	25 (3–55)	29 (5–49)	25 (5–55)	19 (3–52)	0.333			
MMSE	29 (16–30)	29 (27–30)	29 (27–30)	25 (16–26)	**<0.001**	**0.046**	**<0.001**	**<0.001**
ACE-R	87 (60–99)	93 (80–98)	87 (72–99)	79 (60–87)	**<0.001**	**0.001**	**0.020**	**<0.001**
ACE-R AO	18 (13–18)	18 (17-18)	17 (15–18)	15 (13–18)	**<0.001**	0.130	**<0.001**	**<0.001**
ACE-R M	21 (3–26)	23 (17–26)	19 (12–26)	18 (3–21)	**0.001**	0.057	0.126	**0.001**
ACE-R F	11 (3–14)	13 (6–14)	10 (3–14)	9 (5–12)	**0.001**	**0.005**	0.782	**0.004**
ACE-R L	25 (20–26)	26 (24–26)	25 (20–26)	25 (21–26)	0.176			
ACE-R VA	15 (10–16)	16 (13–16)	15 (12–16)	14 (10–16)	**0.003**	0.250	**0.031**	**0.005**

UPDRS III: Unified Parkinson's Disease Rating Scale.

MMSE: Mini Mental State Examination.

ACE-R: Addenbrooke's Cognitive Examination, global score.

ACE-R AO: Addenbrooke's Cognitive Examination, domain attention and orientation.

ACE-R M: Addenbrooke's Cognitive Examination, domain memory.

ACE-R F: Addenbrooke's Cognitive Examination, domain verbal fluency.

ACE-R L: Addenbrooke's Cognitive Examination, domain language.

ACE-R VA: Addenbrooke's Cognitive Examination, domain visual spatial abilities.

**Table 2:** AUC estimates calculated in ROC analyses and ROC characteristics at optimal cut-offs.

	AUC (95% CI)	*p* value	Cut-off	Sensitivity (95% CI)	Specificity (95% CI)	PPV	NPV
ACE-R							
PD-NC versus PD-MCI	0.78 (0.66–0.90)	**<0.001**	88.5	0.68 (0.50–0.81)	0.91 (0.69–0.98)	0.93	0.63
PD-MCI versus PD-D	0.78 (0.63–0.93)	**0.007**	82.5	0.70 (0.35–0.92)	0.73 (0.56–0.86)	0.41	0.90
ACE-R AO							
PD-NC versus PD-MCI	0.64 (0.50–0.78)	0.077	17.5	0.51 (0.35–0.68)	0.73 (0.50–0.88)	0.76	0.47
PD-MCI versus PD-D	0.92 (0.77–1.00)	**<0.001**	15.5	0.90 (0.54–0.99)	0.97 (0.84–1.00)	0.90	0.97
ACE-R M							
PD-NC versus PD-MCI	0.68 (0.55–0.82)	**0.020**	22.5	0.76 (0.58–0.88)	0.50 (0.29–0.71)	0.72	0.55
PD-MCI versus PD-D	0.71 (0.54–0.88)	**0.043**	20.5	0.90 (0.54–0.99)	0.46 (0.30–0.63)	0.31	0.94
ACE-R F							
PD-NC versus PD-MCI	0.75 (0.61–0.88)	**0.002**	11.5	0.70 (0.53–0.84)	0.73 (0.50–0.88)	0.81	0.59
PD-MCI versus PD-D	0.62 (0.42–0.82)	0.264	8.5	0.50 (0.20–0.80)	0.76 (0.58–0.88)	0.36	0.85
ACE-R L							
PD-NC versus PD-MCI	0.62 (0.47–0.76)	0.141	24.5	0.35 (0.21–0.53)	0.91 (0.69–0.98)	0.87	0.45
PD-MCI versus PD-D	0.56 (0.34–0.77)	0.585	23.5	0.40 (0.14–0.73)	0.84 (0.67–0.93)	0.40	0.84
ACE-R VA							
PD-NC versus PD-MCI	0.63 (0.48–0.77)	0.110	15.5	0.62 (0.45–0.77)	0.64 (0.41–0.82)	0.74	0.50
PD-MCI versus PD-D	0.75 (0.58–0.93)	**0.015**	14.5	0.60 (0.27–0.86)	0.78 (0.61–0.90)	0.43	0.88

ACE-R: Addenbrooke's Cognitive Examination, global score.

PD-NC: subjects with Parkinson's disease with normal control.

PD-MCI: subjects with Parkinson's disease with mild cognitive impairment.

PD-D: subjects with Parkinson's disease with dementia.

ACE-R AO: Addenbrooke's Cognitive Examination, domain attention and orientation.

ACE-R M: Addenbrooke's Cognitive Examination, domain memory.

ACE-R F: Addenbrooke's Cognitive Examination, domain verbal fluency.

ACE-R L: Addenbrooke's Cognitive Examination, domain language.

ACE-R VA: Addenbrooke's Cognitive Examination, domain visual spatial abilities.

**Table 3:** Correlation coefficients between ACE-R subscores and relevant neuropsychological tests.

	ACE-R AO	ACE-R M	ACE-R L	ACE-R F	ACE-R VA
WAIS-R C	0.242				
WAIS-R A	0.423^**∗**^				
WAIS-R DS	0.311^**∗**^				
SCWT A	0.331^**∗**^				
SCWT B	**0.289**				
LMI		0.491^**∗**^			
LMD		0.408^**∗**^			
LWI		0.476^**∗**^			
LWD		0.513^**∗**^			
LWR		0.483^**∗**^			
MAST			0.109		
VF			0.194		
WAIS-R S				0.423^**∗**^	
SCWT C				0.545^**∗**^	
WAIS-R PC					0.544^**∗**^
CT					0.758^**∗**^

Statistically significant correlation coefficients at *α* = 0.01 level are marked with bold and “*∗*” symbol; *α* = 0.05 level is marked with bold only.

WAIS-R C: Coding subtest (WAIS-R); WAIS-R A: Arithmetic subtest (WAIS-R).

WAIS-R DS: Digit Span subtest (WAIS-R), SCWT A: Stroop Colour and Word Test—words part, SCWT B: Stroop Colour and Word Test—colours part, LMI: logical memory subtest—immediate (WMS-III), LMD: logical memory subtest—delayed recall (WMS-III), LWI: list of words—immediate (WMS-III), LWD: list of words—delayed (WMS-III), LWR: list of words—recognition (WMS-III), MAST: Mississippi Aphasia Screening Test, VF: verbal fluency—letter (n, k, and p), WAIS-R S: Similarities subtest (WAIS-R), SCWT C: colours in Stroop Colour and Word test—colour and word part, WAIS-R PC: Picture Completion subtest (WAIS-R), and CT: Clock Test.
